# The relations between socio-demographic information and negative symptoms, mental health, and quality of life: a latent profile analysis with psychotic patients in Kosovo

**DOI:** 10.3389/fpsyt.2023.1135385

**Published:** 2023-07-26

**Authors:** Fitim Uka, Jon Konjufca, Fjolla Ramadani, Aliriza Arënliu, Dashamir Bërxulli, Nikolina Jovanović, Manuela Russo

**Affiliations:** ^1^Department of Psychology, University of Pristina “Hasan Prishtina”, Prishtina, Kosovo; ^2^Unit for Social and Community Psychiatry, Queen Mary University of London, London, United Kingdom

**Keywords:** psychosis, socio-demographic information, negative symptoms, mental health, latent profile analysis (LPA), Kosovo

## Abstract

The current study aims to identify meaningful psychotic patients’ profiles by examining certain combinations of patient’s demographic and socio-economic variables (sex, age, marital status, number of children, cohabitant and level of education). Moreover, we aim to assess whether there is any significant effect of class membership (profile) on negative symptoms, health state, and quality of life among psychotic patients. A convenience sample of 103 patients (age: *M = 22*, *SD* = 1.75), was drawn from the clinical populations of Kosovo. Demographic and socio-economic data was obtained through individual interviews, meanwhile a battery of questionnaires was used to assess negative symptoms, mental health, and quality of life of patients. The 4-class solution was selected as the best fitting model and used in subsequent analyses. Results indicated a significant effect of class membership on health state, quality of life and negative symptoms. Practical implications are discussed.

## 1. Introduction

Extensive research has focused on symptoms of patients with psychosis. Because of their nature, the most well studied symptoms have been positive symptoms. However, since empirical evidence shows that negative symptoms are more persistent, a lot of researchers have also investigated the impact that these symptoms have on a range of aspects (1–3). Negative symptoms impact the ability to establish and maintain relationships and to respond well to social situations, by avoiding social interactions and withdrawing from social life (4, 5). It is known that negative symptoms also affect the occupational component, work performance, engagement in household activities, and satisfaction with recreational activities (6–8).

Negative symptoms are an integral part of quality of life in patients with psychosis (9). Quality of life has many definitions mainly due to the lack of consensus among health professionals and clinicians. However, for the purpose of this study, we will use the subjective orientation of quality of life, which involves physical health and social and emotional aspects (10, 11). Patients with psychosis have continuously shown lower levels of quality of life, even when compared with people with other mental health diagnosis (12–14). Distressing symptoms, lack of control, lack of energy and hopelessness and poor social relationships are the crucial domains of quality-of-life experiences in people with psychotic disorders (15). Dimensions of quality of life seem to resemble health status, however these are two different and distinguishable aspects (16). Functional mobility, self-care abilities and other physical aspects of health and mental health are limited in patients with schizophrenia and other non-affective disorders (17–19).

Patients with psychosis differ in the levels of quality of life, negative symptoms and health state depending on several socio-demographic variables. To start with, there are sex differences between psychotic patients as pertains to their health status. Generally, women are shown to perform better in some cognitive tasks as compared to men. Further, a lower level of physical activity has been observed in men relative to women (20, 21). Moreover, sex has also been a predictive variable regarding quality of life. Lower quality of life has been associated with the men (22), but women have also been linked with lower physical quality of life (23). Regarding negative symptoms, results have shown that men experience them more than woman (24). However, there are contradictory results in this case too (25).

Age is another factor that makes the difference regarding the aforementioned variables. Even though self-reported mobility limitations have been prevalent even in younger ages of this population, older people have shown poorer abilities in mobility both on self-reported measures and performance tests (26–28). Conversely, negative symptoms are linked with a younger age (29) However, there is evidence that older age has been linked with more severe negative symptoms, but only in men (30). On the other hand, inconsistent results are also shown in different studies that investigated the relationship between age and quality of life (26).

Marital status has been considered as an aspect that impacts many life domains of people with psychosis, even on the age on-set of the first episode. Being married seems to be a protective factor, as married patients with psychosis report higher quality of life on all its domains, as opposed to single patients (22, 31, 32). In this sense, it is only logical that negative symptoms are more apparent in people who are single (26, 33). It is important to also mention that gender together with marital status are the significant factors on all the aforementioned domains, with single males having lower quality of life and present more negative symptoms (29). However, it has to be noted that negative symptoms have been linked to an increased avoidance of social contact, which may make it difficult to develop relationships (34). On the other hand, single marital status has been linked to psychomotor poverty and disorganization (35). Patients with psychosis who are cohabitating have similar results as married patients in respect of quality of life. Patients who cohabitate have lower levels of suicidal ideation, suggesting an improved mental health state. In addition, cohabitation has been linked to higher functioning (36–38).

An interesting aspect for researchers focused on psychotic patients has been parenting. Even though a proportion of patients with psychosis have children, women more so than men, the distribution depends on the type of psychotic disorder. Patients diagnosed with schizophrenia in general have fewer children than other groups (39). Patients who have children usually may be more motivated to take care of themselves and lead a healthier lifestyle, which may positively impact health state and quality of life. However, stressors related to parental life may worsen the symptoms. In fact, most of the parents with psychotic disorders need and ask for help from other people, usually relatives and friends. However, patients with psychosis who are parents regarding clinical aspects and functions are similar with patients that do not have children. Nevertheless, they have more contact with family members and friends which may help the quality of life, especially the social aspect of it (40–42).

Education level is a socio-economic variable that impacts various aspects of life of patients with psychotic disorders. Patients with a higher level of education report a better quality of life than other patients (26, 43). Benefits of education level on health state have been stated on numerous research studies. Education impacts behaviors related to health which impacts the health state. The higher the level of education, the more people are likely to lead a healthier life that excludes smoking, drinking too much or using substances that are not legal (44). The case stays the same for mental health as well, with more educated people reporting less anxious and depressive symptoms. On the other hand, literature consists of mixed results regarding relationship of level of education and negative symptoms (45, 46).

It is understood that all the aforementioned dependent variables serve as important and protective/risk factors for the quality of life, negative symptoms and health state of patients with psychosis. However, it can also be implied that each alone may not be sufficient to explain the differences between patients with psychosis. In this case, an approach that would be beyond useful is person-centered approach. Previous studies using variable centered approach have limited the conclusion to simple associations among demographic variables and mental health (47). However, each patient has a gender, age, family socio-economic status and education, which is different from other patients. Also, each patient interacts with others in a certain way, which is unique. Most importantly, this interaction takes place in a unique context. As such, focusing only on these multiple dimensions, without studying the intersections and ignoring the context may lead to superficial understanding of the relations between demographic variables and mental health.

By taking a person-centered approach, the present study acknowledges that there might be intra-individual variation in the configuration within the set of selected demographic and socio-demographic variables (sex, age, marital status, number of children, cohabitant and level of education), which may have different effects on negative symptoms, health state and quality of life among the psychotic patients. We chose the person-centered approach, since it may be more informative compared to variable center approach. The person-centered approach does not only provide information about how individuals differ with regard to the selected individual and environmental variables but also shows how the specific combinations of these variables may operate within individuals (48). As such, the current study aims to identify the best class solution by examining certain combinations of patient’s demographic and socio-economic variables (sex, age, marital status, number of children, cohabitant and level of education). After identifying the classes, the next step would be to assess whether there is any significant effect of class membership on negative symptoms, health state and quality of life among the psychotic patients. Since there is no previous study with similar research question, then we did not formulate specific hypotheses but viewed this part of the analyses as exploratory.

## 2. Method

### 2.1. Participants

A convenience sample consisted of 103 patients (age: *M = 22*, *SD* = 1.75), and was drawn from the clinical populations of two Community Mental Health Centers (CMHC) in Pristina and Ferizaj (Kosovo) and from one integrated community housing in Ferizaj. Five criteria were considered for the inclusion of the patients in the present study: (a) being at least 18 years of age; (b) being in a psychiatric treatment for at least 3 months; c) having a clinical diagnosis of psychosis or related disorder (i.e.: ICD-10 F20-29, F31); (d) were not planned to be discharged from mental health services for the next 3 months; and (e) being capable to give informed consent. Most participants were men (*N* = 73, 68%) and half of them (49%) reported elementary school as their highest level of education. Most of the patients were unemployed (*N* = 95, 90%). Also, most of them (61.3%) were single, divorced or widowed and only 33% were in a relationship or married. In total, 102 patients were of Albanian ethnicity, and one was of Ashkali ethnicity, but spoke Albanian fluently.

A total of 40 participants had prior history of hospitalizations, with most participants (24) having only one hospitalization episode. Few participants had two or three prior hospitalizations (six and three participants, respectively), and only a small minority were hospitalized more often. In the last 6 months, only 17 clients had been received in hospitals as inpatients, where 12 of them did so because of psychiatric reasons. A total of 21 respondents reported taking fluphenazine depot medication, whereas only nine received haloperidol depot as part of their treatment.

### 2.2. Procedure

The current study is a part of the EC-funded IMPULSE project (grant number *779334*), which aims to offer a unique opportunity to radically improve the care of people with psychotic disorders in several Southeastern European Countries. All patients who agreed to be involved in this study met with researchers who double-checked if patients met the eligibility criteria. Ethic approval was obtained by the local ethics committee and all participants signed the informed consent before taking part in the study. All researchers were trained in administering the measures, which were translated into the Albanian language. The patients were interviewed individually during a weekday in a quiet area in their Community Mental Health Center. The assessment took approximately 1 h per patient.

### 2.3. Measures

*Demographic and socio-economic data.* Data about demographic and socio-economic characteristics were collected during individual interviews. Information about sex, age, marital status, number of children, cohabitant and level of education, was obtained with an *ad-hoc* researcher-administered questionnaire.

*Clinical Assessment Interview for Negative Symptoms (CAINS)* (49) was used to assesses the severity of five negative symptoms: asociality, avolition, anhedonia, affective flattening, and alogia. Each item (i.e., Motivation for Close Family/Spouse/Partner Relationships) is scored on a 5-point scale ranging from symptoms being absent (0) to severe (4). CAINS contain in total 13 items, which are divided into two scales: Motivation and Pleasure scale (nine items) and Expression (four items). Higher scores reflect greater impairment.

*EQ-5D-5L* (50), was used to assess mobility, self-care, usual activities, pain/discomfort and anxiety/depression. Each dimension has 5 levels ranging from (0) no problems to (4) extreme problems. The digits for the five dimensions can be combined into a 5-digit number that describes the patient’s health state. Higher scores reflect lower levels of health.

*The Recovering Quality of Life* (ReQoL) (51). is a new generic self-reported outcome, which is used to assess the quality of life for people with different mental health conditions. ReQoL has two versions: a brief 10-item measure (ReQoL-10), and a 20-item measure (ReQoL-20). ReQoL-10 contain positively and negatively worded items covering seven themes: activity, hope, belonging and relationships, self-perception, well-being, autonomy, and physical health.

### 2.4. Statistical analysis

First, we explored the descriptive statistics for each of the variables included in this study. Second, we used the Latent Profile Analysis (LPA) to identify homogeneous subgroups of patients with similar patterns of demographic and socio-economic data. The LPA estimates an individual’s probability of membership in each latent class. Several LPA models were estimated, starting with a one-class solution and adding an additional class in each successive model. We compared the absolute values for the Akaike’s information criterion (AIC; lower values are preferable) and Bayesian information criterion (BIC; lower values are preferable). In addition, we also considered the relative decrease in these values over successive models. Second, ANOVA was used to identify whether there is any significant effect of class membership on negative symptoms, health state and quality of life of psychotic patients.

## 3. Results

In total, six different class solutions were tested and compared. Model fit for the one- through six-class solution is presented in Table 1. Entropy values for each solution were above 0.90, indicating adequate classification. The lowest values of AIC, BIC, and a-BIC were found in the 4-class solution. The 4-class solution was selected as the best fitting model and used in subsequent analyses. The four-class model estimated average latent class membership ranged between, which indicated good classification quality. The smallest class included 7 patients.

**Table 1:** Fit statistics for LPA models.

Number of classes	Free parameters	Log-likelihood	BIC	ABIC	AIC	Entropy	Number of patients in the smallest class
1 class	14	−1015.487	2095.975	2051.637	2058.975	---	---
2 class	26	−947.120	2014.743	1932.613	1946.240	0.911	32
3 class	38	−926.010	2028.139	1908.104	1928.019	0.927	8
4 class	50	−892.837	2017.410	1859.469	1885.673	0.941	7
5 class	62	−883.853	2055.059	1879.212	1891.706	0.976	8
6 class	74	−874.637	2092.243	1858.490	1897.273	0.966	1

### 3.1. Exploring class membership

The composition of each class is presented in Table 1. The oldest participants, with highest number of children, highest number of cohabitants, highest number of males and highest number of participants with elementary school as major qualification are in class 2. The youngest participants, with the lowest number of children and cohabitants, and the highest percentage of participants being single are placed in class 3. The class 1 represents a mean age of participants, with highest number of male participants, with highest percentage of divorced participants, and with highest percentage of participants with highest school as highest level of their education. Class 4 represents another profile of patients, with the highest percentage of patients with university level as their highest level of education, with most of the participants being married and with other variables being in the mean level.

### 3.2. The effect of class membership on health state

An analysis of variance showed that the effect of class membership on **mobility** was significant, *F* (3, 99) = 4.27, *p* = 0.007. The mean (*M* = 1.7) of the first class is significantly higher than all other classes. Also, the effect of class membership on **self-care** was only marginally significant, F (3, 99) = 2.51, *p* = 0.063. There were significant differences between class one (M = 1.5) and class three. The effect of class membership on other dimensions of health state, such as activities, pain and anxiety, was not significant (see Table 2).

**Table 2:** Composition of each class.

	Class 1	Class 2	Class 3	Class 4
Age	48.71 (8.13)	54.40 (6.90)	38.33 (8.01)	49.02 (7.16)
Children	1.95 (0.74)	7.14 (1.06)	0.07 (0.26)	3.56 (0.70)
Cohabiting	1.71 (0.72)	2.85 (0.69)	1.67 (0.66)	2.61 (0.69)
**Gender**				
Female	38.1	28.6	29.8	33.3
Male	61.9	71.4	70.2	66.7
**Marital status**				
Single	4.8	0	75.4	0
Married	42.9	85.7	10.5	94.4
Divorced	52.4	14.3	14.0	0
Widowed	0	0	0.00	5.6
**Education**				
Less than elementary school	0	14.3	10.5	0
Elemnetary school	38.1	42.9	33.3	38.9
High school	61.9	28.6	49.1	50.0
University	0	0	7.0	11.1
Professional education	0	0	0	0
Other qualification	0	14.3	0	0

### 3.3. The effect of class membership on quality of life

Results also indicated significant differences between classes regarding the Recovering Quality of Life. There was a significant effect of class membership on the overall scale, *F* (3, 99) = 3.19, *p* = 0.027. Further investigation indicated that class one has a significantly higher mean (M = 16) than class three (6.1) and marginally significant compared to class two (*M* = 8.5) (see Table 3).

**Table 3:** The effect of class membership on health state, quality of life and negative symptoms.

	Class 1		Class 2		Class 3		Class 4		DIFFERENCES
	M	SD	M	SD	M	SD	M	SD	
CAINS Motivation and Pleasure	13.35	6.27	22.28	10.20	17.13	7.23	17.50	6.00	Class 1 < Class 2
CAINS Experience	4.33	3.45	6.28	3.19	5.01	3.90	5.50	3.50	
Mobility	1.71	1.14	0.42	1.13	0.94	1.14	0.66	0.76	Class 1 > Class 2, Class 3, Class 4
Self-care	1.52	0.87	1.42	1.27	1.01	0.71	1.05	0.63	Class 1 > Class 3
Activities	1.57	0.74	1.42	1.13	1.50	1.11	1.33	0.84	
Pain	2.19	1.12	2.42	1.71	1.78	1.34	1.50	0.98	
Anxiety	2.28	1.10	2.00	1.82	1.84	1.43	1.55	1.04	
Quality of life	16.00	12.74	0.85	2.26	6.14	15.05	8.50	14.70	Class 1 > Class 2, Class 3

### 3.4. The effect of class membership on negative symptoms

However, results showed a significant effect of class membership on the dimension of Motivation and Pleasure scale of Negative symptoms *F* (3, 03) = 10.51, *p* = 0.033. Group 1 had significantly lower mean (*M* = 13.5) compared to group two (22.28). However, there was no significant effect of class membership on the dimension of Expression of negative symptoms.

## 4. Discussion

Schizophrenia is a heterogeneous condition which has often resisted efforts to delineate homogeneous subgroups which refer to specific psychosis subtypes. Latent profile analysis is ideally suited to the task of finding groups of patients which are characterized by several demographic variables. This study aimed to identify meaningful psychotic patients’ profiles by examining certain combinations of patient’s demographic and socio-economic variables. Additionally, it intended to see if there is any significant effect of class membership (profile) on negative symptoms, health state and quality of life among the psychotic patients.

To the knowledge of the authors, this study is one of the few which have used exclusively socio-economic and demographic variables in order to define subtypes, in addition, it is one of the few studies which explicitly compare clinically-relevant measurements, such as CAINS, EQ-5D-5L, and ReQoL-10. The results revealed significant differences between different classes pertaining to motivation and pleasure, mobility, self-care, and quality of life.

In previous studies, latent profile analysis was used to classify patients starting from the times of reaching specific developmental milestones during infancy and used the resulting four classes (early, regular, late, and extra late developers) to determine the age of schizophrenia onset. Participants who reached developmental milestones later had the highest cumulative incidence (2.39%), while people with regular development had an intermediate incidence (1.25%) and early developers had the lowest incidence (0.99%) (52). In the present study, age was used as a developmental indicator to distinguish between the four classes.

In addition, latent profile analysis was used to classify psychotic patients into four latent attachment classes according to their scores in an attachment questionnaire (secure, insecure anxious, insecure-avoidant and disorganized) (53). The study showed that most patients exhibited a secure attachment style, whereas a few who displayed a disorganized attachment style were associated with a higher prevalence of physical and sexual abuse, as well as more severe positive symptoms as compared to other attachment classes. In the present study, the attachment proxies which we used to delineate classes were the number of children, as well as the cohabitation and marital status.

Patients belonging to the four classes have shown significant differences in self-care behavior. Latent profile analysis has been used to identify three health literacy profiles among psychotic patients (low, moderate, and high) (54). Compared to other populations in the study, the patient sample showed lower health literacy scores, serving as an indication that self-care may be used as a socio-demographic variable that increases class specificity.

This study provides evidence of the interaction between different demographic variables, which can ease the lifestyle of patients with schizophrenia. When exploring the class membership and the differences in several variables, Class 1 was mostly differing from other classes. In its composition, Class 1 has the highest number of male participants, with highest percentage of divorced participants, and with highest percentage of participants with highest school as their main education. This group scored higher on several domains, indicating better quality of life, self-care, mobility as well as motivation and pleasure. Class 1 showed significantly lower motivation and pleasure when compared to Class 2. Analyzing their composition, it is evident that Class 2 has the oldest participants, with highest number of children, highest number of cohabitants, highest number of males and highest number of participants with elementary school as major qualification and combination of these variables lead to higher motivation and pleasure compared to other characteristics that can be found on Class 1. On the other hand, the Class 3 (the youngest participants, with the lowest number of children and cohabitants, and the highest percentage of participants being single) showed lower mobility, self-care and quality of life compared to Class 1. Also, Class 4 (with the highest percentage of patients with university level as their highest level of education, with most of the participants being married and with other variables being in the mean level) showed lower levels of mobility compared to Class 1. However, there were no significant differences when classes were compared in relation to anxiety, pain and activities.

The study has important implications for healthcare professionals and policymakers, as it highlights the need for tailored interventions to improve the quality of life for different demographic groups. However, future studies are needed, in order to inform policymakers on how to prioritize funding for education programs that target patients at risk. Overall, at can be concluded that personalized interventions and policy solutions are necessary to address the diverse needs of different demographic groups.

This study has several limitations. First, it is unclear whether the schizophrenia subtypes defined here lie in a continuum of severity or if they represent qualitatively different types of psychosis. Second, the smallest identified class consisted of seven patients, making it too small to conduct meaningful inferential analysis in most cases. Third, the classes which were identified refer only to a cross-sectional sample and have not yet been confirmed in an independent sample of psychosis patients. Finally, the classes have been defined in a snapshot sample, but their validity has not been established in a retest of the same sample.

## Data availability statement

The original contributions presented in the study are included in the article/Supplementary material, further inquiries can be directed to the corresponding author.

## Ethics statement

The studies involving human participants were reviewed and approved by Hospital and University Clinical Service of Kosovo – Ethics Committee 2019–85. The patients/participants provided their written informed consent to participate in this study.

## Author contributions

FU, JK, FR, AA, DB, NJ, and MR contributed to the study conception and design. FU, JK, and FR contributed to the acquisition of data. FU contributed to the statistical analysis and interpretation of data. FU, FR, and JK contributed to the drafting of the manuscript. AA, DB, NJ, and MR contributed to the critical revision. All authors contributed to the article and approved the submitted version.

## Funding

This study was funded as part of the IMPULSE project under the European Commission’s Horizon 2020 research and innovation program under grant agreement No 779334. The IMPULSE project has received funding through the “Global Alliance for Chronic Diseases (GACD) prevention and management of mental disorders” (SCI-HCO-07-2017) funding call. The funder had no role in the design of the study, data collection, analysis and interpretation of the data or in the writing of the manuscript.

## Conflict of interest

The authors declare that the research was conducted in the absence of any commercial or financial relationships that could be construed as a potential conflict of interest.

## Publisher’s note

All claims expressed in this article are solely those of the authors and do not necessarily represent those of their affiliated organizations, or those of the publisher, the editors and the reviewers. Any product that may be evaluated in this article, or claim that may be made by its manufacturer, is not guaranteed or endorsed by the publisher.
