# Supplementary material for: The relations between socio-demographic information and negative symptoms, mental health, and quality of life: a latent profile analysis with psychotic patients in Kosovo
Source: Front Psychiatry. 2023 Jul 26;14:1135385. doi: 10.3389/fpsyt.2023.1135385 (PMC10410071; doi:10.3389/fpsyt.2023.1135385)
Supplement: Supplementary file 1 [file Table_1.DOCX]

***Supplementary Material***

**The relations between socio-demographic information and negative symptoms, mental health and quality of life: A latent profile analysis with psychotic patients in Kosovo**

**Fitim Uka, Jon Konjufca, Fjolla Ramadani**^*^**, Aliriza Arënliu, Dashamir Bërxulli, Nikolina Jovanović, Manuela Russo**

*** Correspondence:** Fjolla Ramadani: ffjollaramadani@gmail.com

Table 1. Fit statistics for LPA models

| Number of classes | Free parameters | Log-likelihood | BIC | ABIC | AIC | Entropy | Number of patients in the smallest class |
| --- | --- | --- | --- | --- | --- | --- | --- |
| 1 class | 14 | -1015.487 | 2095.975 | 2051.637 | 2058.975 | --- | --- |
| 2 class | 26 | -947.120 | 2014.743 | 1932.613 | 1946.240 | .911 | 32 |
| 3 class | 38 | -926.010 | 2028.139 | 1908.104 | 1928.019 | .927 | 8 |
| 4 class | 50 | -892.837 | 2017.410 | 1859.469 | 1885.673 | .941 | 7 |
| 5 class | 62 | -883.853 | 2055.059 | 1879.212 | 1891.706 | .976 | 8 |
| 6 class | 74 | -874.637 | 2092.243 | 1858.490 | 1897.273 | .966 | 1 |
