# Supplementary material for: The relations between socio-demographic information and negative symptoms, mental health, and quality of life: a latent profile analysis with psychotic patients in Kosovo
Source: Front Psychiatry. 2023 Jul 26;14:1135385. doi: 10.3389/fpsyt.2023.1135385 (PMC10410071; doi:10.3389/fpsyt.2023.1135385)
Supplement: Supplementary file 2 [file Table_2.DOCX]

***Supplementary Material***

**The relations between socio-demographic information and negative symptoms, mental health and quality of life: A latent profile analysis with psychotic patients in Kosovo**

**Fitim Uka, Jon Konjufca, Fjolla Ramadani**^*^**, Aliriza Arënliu, Dashamir Bërxulli, Nikolina Jovanović, Manuela Russo**

*** Correspondence:** Fjolla Ramadani: [ffjollaramadani@gmail.com](mailto:ffjollaramadani@gmail.com)

Table 2. Composition of each class

|  | Class 1 | Class 2 | Class 3 | Class 4 |
| --- | --- | --- | --- | --- |
| Age | 48.71 (8.13) | 54.40 (6.90) | 38.33 (8.01) | 49.02 (7.16) |
| Children | 1.95 (.74) | 7.14 (1.06) | .07 (.26) | 3.56 (.70) |
| Cohabiting | 1.71 (.72) | 2.85 (.69) | 1.67 (.66) | 2.61 (.69) |
| Gender |  |  |  |  |
| Female | 38.1 | 28.6 | 29.8 | 33.3 |
| Male | 61.9 | 71.4 | 70.2 | 66.7 |
| Marital status |  |  |  |  |
| Single | 4.8 | 0 | 75.4 | 0 |
| Married | 42.9 | 85.7 | 10.5 | 94.4 |
| Divorced | 52.4 | 14.3 | 14.0 | 0 |
| Widowed | 0 | 0 | 0.00 | 5.6 |
| Education |  |  |  |  |
| Less than elementary school | 0 | 14.3 | 10.5 | 0 |
| Elemnetary school | 38.1 | 42.9 | 33.3 | 38.9 |
| High school | 61.9 | 28.6 | 49.1 | 50.0 |
| University | 0 | 0 | 7.0 | 11.1 |
| Professional education | 0 | 0 | 0 | 0 |
| Other qualification | 0 | 14.3 | 0 | 0 |
