# Supplementary material for: The relations between socio-demographic information and negative symptoms, mental health, and quality of life: a latent profile analysis with psychotic patients in Kosovo
Source: Front Psychiatry. 2023 Jul 26;14:1135385. doi: 10.3389/fpsyt.2023.1135385 (PMC10410071; doi:10.3389/fpsyt.2023.1135385)
Supplement: Supplementary file 3 [file Table_3.DOCX]

***Supplementary Material***

**The relations between socio-demographic information and negative symptoms, mental health and quality of life: A latent profile analysis with psychotic patients in Kosovo**

**Fitim Uka, Jon Konjufca, Fjolla Ramadani**^*^**, Aliriza Arënliu, Dashamir Bërxulli, Nikolina Jovanović, Manuela Russo**

*** Correspondence:** Fjolla Ramadani: [ffjollaramadani@gmail.com](mailto:ffjollaramadani@gmail.com)

Table 3. The effect of class membership on health state, quality of life and negative symptoms

|  | Class 1 | |  | Class 2 | |  | Class 3 | |  | Class 4 | |  | DIFFERENCES |
| --- | --- | --- | --- | --- | --- | --- | --- | --- | --- | --- | --- | --- | --- |
|  | M | SD |  | M | SD |  | M | SD |  | M | SD |  |  |
| CAINS Motivation and Pleasure | 13.35 | 6.27 |  | 22.28 | 10.20 |  | 17.13 | 7.23 |  | 17.50 | 6.00 |  | Class 1 < Class 2 |
| CAINS Experience | 4.33 | 3.45 |  | 6.28 | 3.19 |  | 5.01 | 3.90 |  | 5.50 | 3.50 |  |  |
| Mobility | 1.71 | 1.14 |  | .42 | 1.13 |  | .94 | 1.14 |  | .66 | .76 |  | Class 1 > Class 2, Class 3, Class 4 |
| Self-care | 1.52 | .87 |  | 1.42 | 1.27 |  | 1.01 | .71 |  | 1.05 | .63 |  | Class 1 > Class 3 |
| Activities | 1.57 | .74 |  | 1.42 | 1.13 |  | 1.50 | 1.11 |  | 1.33 | .84 |  |  |
| Pain | 2.19 | 1.12 |  | 2.42 | 1.71 |  | 1.78 | 1.34 |  | 1.50 | .98 |  |  |
| Anxiety | 2.28 | 1.10 |  | 2.00 | 1.82 |  | 1.84 | 1.43 |  | 1.55 | 1.04 |  |  |
| Quality of life | 16.00 | 12.74 |  | .85 | 2.26 |  | 6.14 | 15.05 |  | 8.50 | 14.70 |  | Class 1 > Class 2, Class 3 |
